# Association between Subjective Well-Being and Frequent Dental Visits in the German Ageing Survey

**DOI:** 10.3390/ijerph17093207

**Published:** 2020-05-05

**Authors:** Richelle Valdez, Ghazal Aarabi, Kristin Spinler, Carolin Walther, Udo Seedorf, Guido Heydecke, Elzbieta Buczak-Stec, Hans-Helmut König, André Hajek

**Affiliations:** 1 Department of Prosthetic Dentistry, Center for Dental and Oral Medicine, University Medical Center Hamburg-Eppendorf, 20246 Hamburg, Germany; r.valdez@uke.de (R.V.); g.aarabi@uke.de (G.A.); kr.spinler@uke.de (K.S.); c.walther@uke.de (C.W.); u.seedorf@uke.de (U.S.); g.heydecke@uke.de (G.H.); 2 Institute of Medical Sociology, Center Psychosocial Medicine, University Medical Center Hamburg-Eppendorf, 20246 Hamburg, Germany; 3 Department of Health Economics and Health Services Research, Hamburg Center for Health Economics, University Medical Center Hamburg-Eppendorf, 20246 Hamburg, Germany; e.buczak-stec@uke.de (E.B.-S.); h.koenig@uke.de (H.-H.K.)

**Keywords:** dentistry, subjective well-being, life satisfaction, affect, dental visits, frequent attender, dental care utilization, dental care use, positive affect, negative affect, affective well-being

## Abstract

The relationship between subjective well-being (SWB) and frequent attendance is understudied. This study used data from a large German sample of non-institutionalized individuals aged 40+ in 2014 (*n* = 7264). SWB was measured using the Satisfaction with Life Scale (SWLS) and the Positive and Negative Affect Schedule (PANAS). Number of self-reported dental visits in the past twelve months was used to measure the utilization frequency of dental services. Individuals with at least four dental visits in the preceding year (highest decile) were defined as frequent dental visits. Robustness checks were performed using alternative cut-offs to define frequent dental visits. Multiple logistic regressions showed that frequent dental visits (highest decile) were associated with less satisfaction with life [OR: 0.89, 95%-CI: 0.80–0.99] and higher negative affect [OR: 1.41, 95%-CI: 1.22–1.64], whereas it was not significantly associated with positive affect. Both associations depended on the cut-off chosen to define frequent dental visits. The present study highlights the association between SWB (particularly negative affect and low life satisfaction) and frequent dental visits. Further studies evaluating patients’ motivation for high dental service use are necessary to check the robustness of our findings.

## 1. Introduction

Previous research has shown that the effects of oral diseases are not restricted to the oral area, but may also play a role in many other diseases that occur in the rest of the body. For example, periodontal disease has been shown to share the same signal molecules and enzymes that control the chronic inflammatory reaction found also in atherosclerotic vascular disease [1,2]. Furthermore, poor oral health has also been observed to have a negative influence on quality of life [3,4]. An example of this is tooth loss, which may result in not only chewing impairments, but also contribute to lower self-esteem (due to aesthetics) [5,6] and communication difficulties [6]. 

Routine dental check-ups have been regarded to be important in preventing and treating oral diseases [7,8]. However, although many countries have the current suggestion to have a dental check up every six months [9], there is still an ongoing debate about what the optimal length of time between dental checkups should be [9,10]. A recent Cochrane review found only one Randomized Controlled Trial (RCT) that tested the effectiveness of six-month dental check-ups [9]. This trial was assessed to be highly biased and the review concluded that there is insufficient evidence to support or refute the benefits of six-month dental check-ups [9]. A new RCT in the UK is currently being conducted to test the efficacy of different dental recall intervals [11].

Shorter lengths of time between dental checks may allow greater opportunities to implement preventive care, as well as catch problems and provide timely therapeutic care [9], while an increased length of time between dental checkups may also delay the diagnosis of dental disease and provision of preventive advice, thus contributing to more extensive and expensive dental care in the long run [9]. This may be true for patients who rarely go to the dentist [12,13] or those who sporadically go when there is a problem [12,14]. 

On the other hand, visiting the dentist too often may increase the chance of overtreatment and iatrogenesis (illness caused by treatment) [9]. This may especially occur in “high service users” (individuals who “excessively” use dental health services).

Although research about the factors that influence the frequency of oral health service is comprehensive, studies specifically about high service users in dentistry are limited [15]. A few studies that took place in Finland undertook such research in adults as a response to the abolishment of a law that restricted adults’ access to public dental services, in which costs had to be paid out of pocket [16,17,18]. Following this abolishment was a 21% increase of dental service utilization in adults from 2000 to 2009 [15]. Using data from this patient register (*n* = 25,993), they discovered that 10.5% were high service users (with “frequent attendance” defined as having made 6+ dental visits/12 months) and their treatment consisted of 31.6% of all adult dental visits [19]. 

Research in primary care about high service users suggests that learning more about high service users is important not only because high service users may incur high costs to the health care system [20,21,22], but also because overly frequent attendance may be an indicator for underlying psychosocial distress [23]. High service use is said to be not just a result of physical disease, but is a representation of the interaction between age, gender, and also psychiatric disorders [23]. Because high service users have also been observed to experience psychiatric comorbidity (e.g., anxiety disorders, somatic symptom disorders) [23,24,25], this may suggest that high service users are a special population that require special evaluation and provision of appropriate psychological treatments [24]. In this way, the frequent health service utilization of high service users could be linked to well-being. 

Based on previous research about high service users in primary care, it might be speculated that the dental service utilization patterns seen in high service users may be influenced by one’s subjective well-being. Subjective well-being (SWB) has been theorized to have two core components: life satisfaction (cognitive well-being–cognitive evaluation of life as a whole) and affective well-being, which is comprised of positive affecting factors (e.g., feelings of happiness and joy) and negative affecting factors (e.g., feelings of sadness and anxiety) [26,27]. Individuals who more frequently experience positive affect, life satisfaction and infrequent negative affect are said to have high SWB [26]. The most common instruments to operationalize SWB include the Satisfaction with Life Scale (SWLS) [28] and the Positive and Negative Affect Schedule (PANAS) [29], which measure the cognitive and affective components of SWB respectively [26]. 

Therefore, the present study aims to examine the association between subjective well-being (SWB) and frequent attendance (in which “frequent attendance” was operationalized using different cut off points for number of dental visits) by using data from a large sample of non-institutionalized individuals who were aged 40+ in Germany in 2014.

## 2. Materials and Methods 

### 2.1. Sample

For this study, cross-sectional data were gathered from the most recent fifth wave (2014) of the German Ageing Survey (DEAS), which is a representative study of community-dwelling individuals in the second half of life (≥40 years). The DEAS study is funded by the Federal Ministry for Family Affairs, Senior Citizens, Women and Youth (BMFSFJ). For instance, data were collected about income, social ties, well-being, and health. Previous waves took place in 1996 (first wave), 2002 (second wave), 2008 (third wave) and 2011 (fourth wave). 

In Wave 5, more than 4000 individuals already took part in previous waves (response rate: 61%) and approximately 6000 individuals (birth cohorts 1929 to 1974) were first time participants (response rate: 25%). Moreover, in the fifth wave, 7264 participants provided data on dental visits. Further details with regard to the DEAS study are provided elsewhere [30]. In our study, the fifth wave was used as we were interested in examining the association between SWB and frequent dental visits and the exact number for all dental visits was only reported in this wave. 

All subjects provided written informed consent. Please note that an ethical statement for the DEAS study was not necessary because the criteria for the need of an ethical statement were not met (risk for the respondents, lack of information about the aims of the study, examination of patients).

The anonymized data sets of the DEAS are available for secondary analysis. The data have been made available to scientists at universities and research institutes exclusively for scientific purposes. The use of data is subject to written data protection agreements. Microdata of the German Ageing Survey (DEAS) are available free of charge to scientific researchers for non-profitable purposes.

### 2.2. Dependent Variable

Frequent dental visits were measured using the self-reported number of dental visits in the preceding 12 months. House calls were included. Only collecting a prescription was not counted as a visit. The exact wording was: “Did you visit one of the following doctors in the past twelve months? If yes, please state how often. Please include house calls. Collecting a prescription is not considered as a visit.”. Among others, individuals were asked about dental visits.

In accordance with numerous previous studies examining frequent doctor visits [31,32], frequent attendance was expected if the number of dental visits in the preceding 12 months was ≥4 (highest decile) in the main analysis. To check whether our findings were robust, highest quartile (≥2 visits) and highest 5% (≥5 visits) were also used as outcome measures in sensitivity analyses. 

### 2.3. Independent Variables: SWB

The well-validated [33] positive and negative affect schedule (PANAS) [5] was used to quantify negative affect and positive affect (each consisting of ten items, ranging from 1 = “very slightly or not at all” to 5 = “extremely”). Individuals were asked about their feelings during the past week. By averaging the score of the corresponding items, the index score for PA and NA (each ranging from 1 to 5) was computed. Higher values correspond to higher positive affect/negative affect. The German version of the PANAS has been validated by Krohne et al [34]. In our study, Cronbach’s alpha for the PA and NA subscales were 87 and 86, respectively. 

Life satisfaction was quantified using the Satisfaction with Life Scale (SWLS) [35]. The SWLS has very good psychometric properties [36], consisting of five items (each ranging from 1 = “strongly agree” to 5 = “strongly disagree”). An index score was computed by averaging the score of the items. Higher values reflect higher satisfaction with life. The German version of the SWLS has recently been validated by Hinz et al [37]. In the current study, Cronbach’s alpha was 86. 

### 2.4. Independent Variables: Control Variables

A set of control variables was included in this study. It was adjusted for sex, age (in years), marital status (single; divorced; widowed; married, living separated from spouse; married, living together with spouse), region (West Germany; East Germany) and income (individual monthly net equivalence income in Euro; new OECD scale). Furthermore, it was adjusted for self-reported Body Mass Index (BMI), smoking status (yes, daily; yes, sometimes; no, not anymore, never been a smoker), days with alcohol consumption (daily; several times a week; once a week; 1 to 3 times a month; less often; never) and the number of physical illnesses (yes or no; hearing problems, ear problems; vision impairment, eye problems; bladder problems; gall bladder, liver or kidney problems; diabetes; cancer; stomach and intestinal problems; respiratory problems, asthma, shortness of breath; joint, bone, spinal or back problems; bad circulation; cardiac and circulatory disorders) which ranges from 0 to 11. 

Some studies have demonstrated that frequent attendance is associated with depression [38,39,40]. For this reason, the main model was extended by adding depression (1 if Center for Epidemiological Studies Depression Scale (15 items, 0–45) ≥ 18 [41]) to test the robustness of our findings. 

In sum, we included as covariates: age, equivalence income, number of chronic diseases, Body-Mass-Index, as well as dummy variables for sex, marital status, region, alcohol consumption and smoking status.

### 2.5. Statistical Analysis

In a first step, bivariate comparisons between non-frequent attenders and frequent attenders were performed (using Chi²-tests and independent *t*-tests, as appropriate). In a second step, multiple logistic regressions were conducted. The criterion for statistical significance was set at *p* < 0.05. Analyses were performed using Stata 15.1 (StataCorp, College Station, TX, USA). 

## 3. Results

### 3.1. Bivariate Associations

Mean dental visits were 2.0 (±2.2; median: 2) in the past 12 months, ranging to 50 visits (12.0% of the individuals reported no dental visits). The distribution of dental visits is also displayed in Figure S1. While the highest quartile had at least two dental visits, the highest decile had ≥ four dental visits and the top 5% had five or more dental visits (results are not shown in Table 1).

Stratified by status (non-frequent dental visits; frequent dental visits), sample characteristics are depicted in Table 1. In comparison to individuals with non-frequent dental visits, high dental service users (highest decile in dental visits) were more often female, and had a higher number of physical illnesses. In addition, high dental service users had a lower satisfaction with life and higher negative affect, whereas it was not associated with positive affect.

### 3.2. Regression Analysis

Unadjusted models (without further covariates) and models simultaneously including life satisfaction, positive and negative affect (with covariates) are included in the Tables S1–S4. The determinants of frequent dental visits are depicted in Table 2 (highest decile; main model). Adjusting for potential confounders, multiple logistic regressions showed that frequent dental visits were associated with less satisfaction with life [OR: 0.89, 95%-CI: 0.80–0.99] and higher negative affect [OR: 1.41, 95%-CI: 1.22–1.64], whereas it was not significantly associated with positive affect. 

When the highest quartile was used as cut-off to define frequent dental visits (Table 3), the association between frequent dental visits and satisfaction with life disappeared, whereas the association between frequent dental visits and positive affect reached statistical significance [OR: 1.11, 95%-CI: 1.01–1.23]. The association between frequent dental visits and negative affect only slightly decreased [OR: 1.27, 95%-CI: 1.15–1.40]. 

When the top 5% was used as cut-off to define frequent dental visits (Table 4), only the association between frequent dental visits and higher negative affect remained statistically significant [OR: 1.35, 95%-CI: 1.12–1.64]. 

Furthermore, models with life satisfaction and positive and negative affect (from the fourth wave, which took place in the year 2011) as the main independent variables are included in Table S5. Thus, we checked whether well-being measures in Wave 4 were associated with frequent dental visits in Wave 5. The findings can be found in detail in Table S5. However, it is worth noting that similar results were obtained.

In further sensitivity analysis, the main model was extended by adding depression to the regression model. In terms of effect sizes and significance, findings remained almost the same (results are not shown, but available upon request). 

## 4. Discussion

### 4.1. Main Findings

Based on a nationally representative sample (individuals ≥ 40 years), the objective of the current study was to examine the association between SWB and frequent attendance. Adjusting for socioeconomic, health-related and lifestyle factors, multiple logistic regressions revealed that frequent dental visits were consistently associated with low SWB (higher negative affect).

### 4.2. Previous Research and Possible Explanations

The association between frequent dental visits and low SWB observed in the present study can be explained by previous literature. Nihtila and colleagues [15,19] observed that, following the dental health care reform in Finland and the subsequent increase of dental service utilization, heavy service users were characterized as needing restorative, endodontic and prosthetic treatment. These reasons for visits were also reflected in the reported oral health status of these heavy service users, who had more caries and periodontal pockets than low service users [19]. A subsequent longitudinal cohort study [15] found that 61.6% of these heavy service users changed to the low user category over a five-year period, while 11.2% persisted in being heavy service users. The most common reason for visits among heavy service users was emergency visits (e.g., repetitive repair or replacement of restorations) in comparison to low users [15]. Logistic regression analyses also showed that chronic heavy dental service use was significantly associated with being 65+ years (reference: 18–29 years), retired (reference: students), and having emergency treatments (reference: no emergency treatments) [15]. In this way, these findings may suggest that the reasons for frequent dental visits may be for curative purposes and not necessarily for preventive care. However, further research is needed in this research area.

These findings align with the sample investigated by the present study: older adults. The sample in the present study may have had poor oral health that required multiple dental visits for prosthetic treatment, perhaps as a result of tooth loss (i.e., dentures, dental bridges, or implants). According to data from the Fifth German Oral Health Survey [7], tooth loss is highly prevalent in the age group of our study. Studies have found that increased number of missing teeth was associated with higher impact on quality of life in terms of perception of handicap, dysfunction, discomfort, and disability [42,43]. Studies investigating psychological aspects of tooth loss have shown that edentulous patients report a lack of self-confidence, altered and inhibited behavior [44,45,46], as well as dissatisfaction with their change in facial appearance. 

Aside from poor oral health, there could be another explanation why frequent dental visits were consistently associated with low SWB. In primary health care research, Karlsson and colleagues profiled different high service users, namely they can be categorized as patients with (1) physical illnesses, (2) psychiatric illness, (3) ongoing life crisis, (4) chronic somatization, and (5) a variety of problems [47]. These high service user profiles may also hold true for the sample of high dental service users in the present study, who may have had lower SWB due to the negative effects of a physical oral illness (e.g., a dental problem requiring extensive treatment), psychiatric illness or somatization. 

Various psychosomatic disorders pertaining to dental practice have been observed [48]. These include pain disorders such as myofascial pain dysfunction syndrome, altered oral sensation disorders such as burning mouth syndrome, and body dysmorphic disorder [48,49]. Such somatization may lead to unreasonable requests for dental treatment and a high number of dental visits or treatments. A previous study investigating the incidence of somatization in general dental practice found that most frequent characteristic of somatization-specific behavior was a remarkably high attendance of dental visits [49]. Those who exhibited somatization-specific behavior also showed significantly higher scores of depression than patients who did not exhibit signs of somatization. In this way, some of our sample may have been experiencing underlying psychosocial distress, resulting in lower SWB and somatization, leading to excessive visits to the dentist. 

Another interesting result of the present study is that although negative affect was consistently associated with frequent dental visits, the three models had additional significant associations. Namely, the association with negative affect seen in the highest quartile model may have been due to oral health problems the participants have been experiencing. The positive affect may be significantly associated with the initial happiness of seeking out and receiving treatment. However, it seems that this pay off of positive affect diminishes with rising visits until only a negative affect is associated with frequent dental visits.

### 4.3. Strengths and Limitations

The strengths of this study are (i) the large sample size, which came from a population-based and thus representative sample of non-institutionalized individuals ≥ 40 years [30], (ii) that SWB was quantified with well-validated and widely used scales, and (iii) that different cut-offs were used to define frequent visits. Sensitivity analysis (different cut-offs) was performed because it is important to test the robustness of our findings. This is also recommended by current guidelines [50]. 

The major limitations are (i) the cross-sectional design, which did not allow for the assessment of the causality and the temporal relationship between SWB and frequent dental visits, (ii) the use of self-reported rather than documented dental visits, which suggested the possibility of a recall bias, and (iii) that neither the reasons for the dental visits nor the treatment procedures and the objective oral health status of the participants had been documented. Furthermore, population-weights were not applied. Thus, we cannot entirely dismiss the possibility that the data may not be representative of the German population. However, it is worth noting that we did not use weights because they can negatively influence the estimates in their efficiency [51]. In addition, a small sample selection bias was demonstrated in the DEAS study [30].

## 5. Conclusions

The present study highlights the association between SWB (in particular, negative affect and low life satisfaction) and frequent dental visits. Further studies are necessary to evaluate patients’ motivation for high dental service use in order to check the robustness of our findings. 

## Figures and Tables

**Table 1:** Sample characteristics, by status (non-frequent dental visits; frequent dental visits (*n* = 7264).

Variables	Non-Frequent Dental Visits (*n* = 6396)	Frequent Dental Visits (*n* = 868)	*p* Value
Female (Ref.: Male): *n* (%)	3211 (50.2)	499 (57.5)	<0.001
Age in years: Mean (SD)	64.4 (11.3)	64.0 (10.4)	0.24
Married, living together with spouse (Ref.: Others): *n* (%)	4503 (70.6)	600 (69.3)	0.45
Monthly net equivalence income in Euro: Mean (SD)	1959.0 (1,408.3)	1893.1 (1154.9)	0.20
East Germany (Ref.: West Germany): *n* (%)	2114 (33.1)	297 (34.2)	0.49
Body-Mass Index (BMI): Mean (SD)	26.9 (4.6)	26.7 (4.5)	0.30
Current smoker (Ref.: No): *n* (%)	1119 (17.6)	148 (17.2)	0.76
Daily alcohol consumption (Ref.: Less than daily alcohol consumption): *n* (%)	783 (12.3)	99 (11.4)	0.47
Number of physical illnesses: Mean (SD)	2.6 (1.9)	2.8 (1.9)	<0.001
Life satisfaction: Mean (SD)	3.8 (0.7)	3.7 (0.8)	<0.01
Positive affect: Mean (SD)	3.6 (0.5)	3.5 (0.5)	0.19
Negative affect: Mean (SD)	2.1 (0.5)	2.2 (0.6)	<0.001

Notes: *n* = number; SD = standard deviation; Comparisons between the two groups were done using *t*-test or chi-square procedures.

**Table 2:** Determinants of frequent dental visits (0 = Non-frequent dental visits; 1 = Frequent dental visits; cut-off at the highest decile). Results of multiple logistic regressions ^1^.

	(1)	(2)	(3)
Independent Variables	Frequent Dental Visits	Frequent Dental Visits	Frequent Dental Visits
Potential confounders	✓	✓	✓
Life satisfaction	0.89 *		
	(0.80–0.99)		
Positive affect		0.95	
		(0.82–1.11)	
Negative affect			1.41 ***
			(1.22–1.64)
Observations	6553	6547	6546
Pseudo R²	0.01	0.01	0.02

^1^ All estimations include age, equivalence income, number of chronic diseases, Body–Mass Index, as well as dummy variables for sex, marital status, region, alcohol consumption and smoking status as potential confounders. Odds ratios were reported; 95% confidence intervals in parentheses; *** *p* < 0.001, * *p* < 0.05.

**Table 3:** Determinants of frequent dental visits (0 = Non-frequent dental visits; 1 = Frequent dental visits; cut-off at the highest quartile). Results of multiple logistic regressions ^1^.

	(1)	(2)	(3)
Independent Variables	Frequent Dental Visits	Frequent Dental Visits	Frequent Dental Visits
Potential confounders	✓	✓	✓
Life satisfaction	1.00		
	(0.93–1.07)		
Positive affect		1.11 *	
		(1.01–1.23)	
Negative affect			1.27 ***
			(1.15–1.40)
Observations	6553	6547	6546
Pseudo R²	0.01	0.01	0.01

^1^ All estimations include age, equivalence income, number of chronic diseases, Body–Mass Index, as well as dummy variables for sex, marital status, region, alcohol consumption and smoking status as potential confounders. Odds ratios were reported; 95% confidence intervals in parentheses; *** *p* < 0.001, * *p* < 0.05.

**Table 4:** Determinants of frequent dental visits (0 = Non-frequent dental visits; 1 = Frequent dental visits; cut-off at the highest 5%). Results of multiple logistic regressions ^1^.

	(1)	(2)	(3)
Independent Variables	Frequent Dental Visits	Frequent Dental Visits	Frequent Dental Visits
Potential confounders	✓	✓	✓
Life satisfaction	0.92		
	(0.80–1.06)		
Positive affect		0.91	
		(0.75–1.10)	
Negative affect			1.35 **
			(1.12–1.64)
Observations	6553	6547	6546
Pseudo R²	0.02	0.02	0.02

^1^ All estimations include age, equivalence income, number of chronic diseases, Body–Mass Index, as well as dummy variables for sex, marital status, region, alcohol consumption and smoking status as potential confounders. Odds ratios were reported; 95% confidence intervals in parentheses; ** *p* < 0.01.

## Supplementary Materials

The following are available online at https://www.mdpi.com/1660-4601/17/9/3207/s1, Figure S1: Distribution of dental visits; Table S1: Determinants of frequent dental visits (0 = Non-frequent dental visits; 1 = Frequent dental visits; cut-off at the highest decile). Results of logistic regressions; Table S2: Determinants of frequent dental visits (0 = Non-frequent dental visits; 1 = Frequent dental visits; cut-off at the highest quartile). Results of logistic regressions; Table S3: Determinants of frequent dental visits (0 = Non-frequent dental visits; 1 = Frequent dental visits; cut-off at the highest 5%). Results of logistic regressions; Table S4: Determinants of frequent dental visits (0 = Non-frequent dental visits; 1 = Frequent dental visits). Results of multiple logistic regressions; Table S5: Determinants of frequent dental visits (0 = Non-frequent dental visits; 1 = Frequent dental visits). Results of multiple logistic regressions.
