# Supplementary material for: Association between Subjective Well-Being and Frequent Dental Visits in the German Ageing Survey
Source: Int J Environ Res Public Health. 2020 May 5;17(9):3207. doi: 10.3390/ijerph17093207 (PMC7246676; doi:10.3390/ijerph17093207)
Supplement: Supplementary file 1 [file ijerph-17-03207-s001.zip › ijerph-766894-supplementary-Final.docx]

**Table S1.** Determinants of frequent dental visits (0 = Non-frequent dental visits; 1 = Frequent dental visits; cut-off at the highest decile). Results of logistic regressions.

|  | **(1)** | **(2)** | **(3)** |
| --- | --- | --- | --- |
| Independent variables | Frequent dental visits | Frequent dental visits | Frequent dental visits |
| Life satisfaction | 0.87 ** |  |  |
|  | (0.79–0.95) |  |  |
| Positive affect |  | 0.91 |  |
|  |  | (0.80–1.05) |  |
| Negative affect |  |  | 1.51 *** |
|  |  |  | (1.32–1.72) |
| Observations | 7205 | 7198 | 7199 |
| Pseudo R² | 0.001 | 0.001 | 0.010 |

Odds ratios were reported; 95% confidence intervals in parentheses; *** *p* < 0.001, ** *p* < 0.01, * *p* < 0.05, + *p* < 0.10.

**Table S2.** Determinants of frequent dental visits (0 = Non-frequent dental visits; 1 = Frequent dental visits; cut-off at the highest quartile). Results of logistic regressions.

|  | **(1)** | **(2)** | **(3)** |
| --- | --- | --- | --- |
| Independent variables | Frequent dental visits | Frequent dental visits | Frequent dental visits |
| Life satisfaction | 1.00 |  |  |
|  | (0.94–1.06) |  |  |
| Positive affect |  | 1.11 * |  |
|  |  | (1.02–1.21) |  |
| Negative affect |  |  | 1.28 *** |
|  |  |  | (1.17–1.40) |
| Observations | 7205 | 7198 | 7199 |
| Pseudo R² | 0.001 | 0.001 | 0.003 |

Odds ratios were reported; 95% confidence intervals in parentheses; *** *p* < 0.001, ** *p* < 0.01, * *p* < 0.05, + *p* < 0.10.

**Table S3**. Determinants of frequent dental visits (0 = Non-frequent dental visits; 1 = Frequent dental visits; cut-off at the highest 5%). Results of logistic regressions.

|  | **(1)** | **(2)** | **(3)** |
| --- | --- | --- | --- |
| Independent variables | Frequent dental visits | Frequent dental visits | Frequent dental visits |
| Life satisfaction | 0.86 * |  |  |
|  | (0.76­0.97) |  |  |
| Positive affect |  | 0.87 |  |
|  |  | (0.73–1.03) |  |
| Negative affect |  |  | 1.54 *** |
|  |  |  | (1.30–1.81) |
| Observations | 7205 | 7198 | 7199 |
| Pseudo R² | 0.002 | 0.001 | 0.007 |

Odds ratios were reported; 95% confidence intervals in parentheses; *** *p* < 0.001, ** *p* < 0.01, * *p* < 0.05, ^+^ *p* < 0.10.

**Table S4**. Determinants of frequent dental visits (0 = Non-frequent dental visits; 1 = Frequent dental visits). Results of multiple logistic regressions.^1^

|  | (1) | (2) | (3) |
| --- | --- | --- | --- |
| Independent variables | Frequent dental visits (cut-off at the highest decile) | Frequent dental visits (cut-off at the highest quartile) | Frequent dental visits (cut-off at the highest 5%) |
| Potential confounders | 🗸 | 🗸 | 🗸 |
|  |  |  |  |
| Life satisfaction | 0.96 | 1.02 | 1.00 |
|  | (0.84–1.08) | (0.94–1.11) | (0.85–1.18) |
| Positive affect | 1.09 | 1.21 *** | 1.00 |
|  | (0.92–1.29) | (1.08–1.35) | (0.80–1.24) |
| Negative affect | 1.42 *** | 1.36 *** | 1.35 ** |
|  | (1.20–1.67) | (1.21–1.51) | (1.10–1.67) |
| Observations | 6541 | 6541 | 6541 |
| Pseudo R² | 0.015 | 0.012 | 0.018 |

^1^ All estimations include age, equivalence income, number of chronic diseases, Body–Mass Index, as well as dummy variables for sex, marital status, region, alcohol consumption and smoking status as potential confounders. Odds ratios were reported; 95% confidence intervals in parentheses; *** *p* < 0.001, ** *p* < 0.01, * *p* < 0.05, ^+^ *p* < 0.10.

**Table S5.** Determinants of frequent dental visits (0 = Non-frequent dental visits; 1 = Frequent dental visits). Results of multiple logistic regressions.^1.^

| Independent variables | Frequent dental visits (Top 10%) | Frequent dental visits (Top 10%) | Frequent dental visits (Top 10%) | Frequent dental visits (Top 25%) | Frequent dental visits (Top 25%) | Frequent dental visits (Top 25%) | Frequent dental visits (Top 5%) | Frequent dental visits (Top 5%) | Frequent dental visits (Top 5%) |
| --- | --- | --- | --- | --- | --- | --- | --- | --- | --- |
| Potential confounders | 🗸 | 🗸 | 🗸 | 🗸 | 🗸 | 🗸 | 🗸 | 🗸 | 🗸 |
| Life satisfaction | 0.92 |  |  | 0.93 |  |  | 0.92 |  |  |
|  | (0.76–1.10) |  |  | (0.82­1.05) |  |  | (0.73–1.16) |  |  |
| Positive affect |  | 0.92 |  |  | 1.13 |  |  | 0.90 |  |
|  |  | (0.72–1.19) |  |  | (0.95–1.35) |  |  | (0.65–1.25) |  |
| Negative affect |  |  | 1.43 ** |  |  | 1.16 |  |  | 1.49 * |
|  |  |  | (1.11–1.85) |  |  | (0.97–1.38) |  |  | (1.08–2.05) |
| Observations | 2303 | 2305 | 2305 | 2303 | 2305 | 2305 | 2303 | 2305 | 2305 |
| Pseudo R² | 0.02 | 0.02 | 0.02 | 0.01 | 0.01 | 0.01 | 0.02 | 0.02 | 0.03 |

^1^ All estimations include age, equivalence income, number of chronic diseases, Body–Mass Index, as well as dummy variables for sex, marital status, region, alcohol consumption and smoking status as potential confounders. Odds ratios were reported; 95% confidence intervals in parentheses; *** *p* < 0.001, ** *p* < 0.01, * *p* < 0.05, + *p* < 0.10.
