# Supplementary figures and images for: Association between Subjective Well-Being and Frequent Dental Visits in the German Ageing Survey
Source: Int J Environ Res Public Health. 2020 May 5;17(9):3207. doi: 10.3390/ijerph17093207 (PMC7246676; doi:10.3390/ijerph17093207)

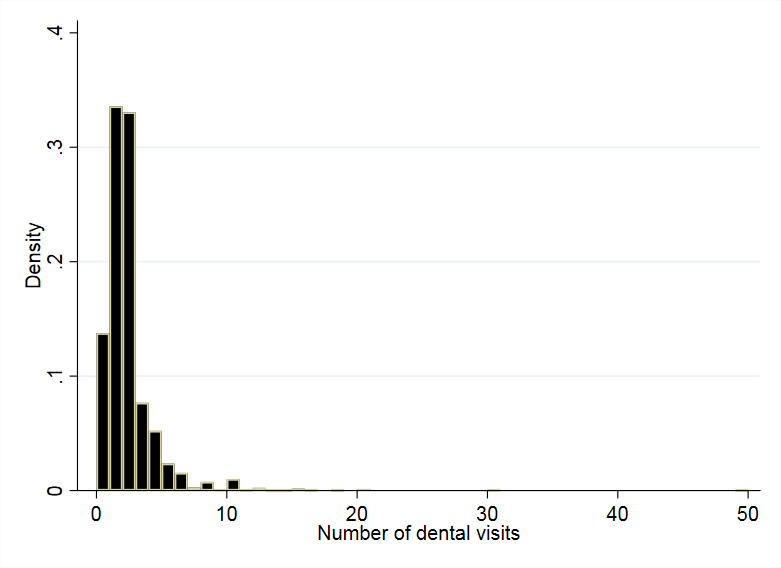

Supplement: Supplementary file 1 [file ijerph-17-03207-s001.zip › Figure S1.tif]
